# *BioXTAS RAW*: improvements to a free open-source program for small-angle X-ray scattering data reduction and analysis

**DOI:** 10.1107/S1600576717011438

**Published:** 2017-09-05

**Authors:** Jesse Bennett Hopkins, Richard E. Gillilan, Soren Skou

**Affiliations:** a Cornell High Energy Synchrotron Source, Ithaca, NY 14853, USA; b SAXSLAB ApS, Denmark

**Keywords:** small-angle X-ray scattering, SAXS, data analysis, size exclusion chromatography, SEC-SAXS, computer programs, *BioXTAS RAW*

## Abstract

*BioXTAS RAW* is a graphical-user-interface-based free open-source Python program for reduction and analysis of small-angle X-ray solution scattering (SAXS) data. The software is designed for biological SAXS data and enables creation and plotting of one-dimensional scattering profiles from two-dimensional detector images, standard data operations such as averaging and subtraction and analysis of radius of gyration and molecular weight, and advanced analysis such as calculation of inverse Fourier transforms and envelopes. It also allows easy processing of inline size-exclusion chromatography coupled SAXS data and data deconvolution using the evolving factor analysis method. It provides an alternative to closed-source programs such as *Primus* and *ScÅtter* for primary data analysis. Because it can calibrate, mask and integrate images it also provides an alternative to synchrotron beamline pipelines that scientists can install on their own computers and use both at home and at the beamline.

## Abstract 2

*BioXTAS RAW* is a graphical-user-interface-based free open-source Python program for reduction and analysis of small-angle X-ray solution scattering (SAXS) data, including size-exclusion chromatography coupled SAXS data. The software is designed for biological data and enables creation and plotting of one-dimensional scattering profiles from two-dimensional detector images, standard data operations such as averaging and subtraction and analysis of radius of gyration and molecular weight, and more advanced analyses such as calculation of inverse Fourier transforms.

## 1. Introduction   

Small-angle X-ray solution scattering (SAXS) is a popular structural technique for studying biological macromolecules. SAXS can provide information about the solution state of macromolecules and complexes, including, but not limited to, size, molecular weight, oligomeric state, flexibility, foldedness and overall shape (Svergun & Koch, 2003 ▸; Jacques & Trewhella, 2010 ▸; Blanchet & Svergun, 2013 ▸; Graewert & Svergun, 2013 ▸; Petoukhov & Svergun, 2013 ▸; Vestergaard & Sayers, 2014 ▸; Chaudhuri, 2015 ▸). Closely tied with the increasing popularity of biological SAXS is the increasing number of computer programs to analyze the data.

SAXS data are primarily acquired on two-dimensional area detectors and then reduced to one-dimensional profiles of scattered intensity *versus* scattering vector magnitude *q* [*q* = 4πsin(θ)/λ, where 2θ is the scattering angle and λ is the X-ray wavelength] (Blanchet & Svergun, 2013 ▸; Pauw, 2013 ▸; Petoukhov & Svergun, 2013 ▸; Dyer *et al.*, 2014 ▸; Skou *et al.*, 2014 ▸). Many programs exist for performing this reduction: stand­alone programs or libraries such as *FIT2D*, *pyFAI* and *DPDAK* (Hammersley *et al.*, 1996 ▸; Benecke *et al.*, 2014 ▸; Ashiotis *et al.*, 2015 ▸; Hammersley, 2016 ▸); custom solutions for a particular synchrotron beamline, such as the version of *Blu-ICE* (McPhillips *et al.*, 2002 ▸) used at BL 4-2 at the SSRL, or home source company, such as the *SAXSLab* software from Rigaku; and, increasingly, data processing pipelines at synchrotron beamlines, such as those at P12 at Petra III and BM29 at the ESRF (Franke *et al.*, 2012 ▸; Blanchet *et al.*, 2015 ▸; Brennich *et al.*, 2016 ▸).

Once obtained, macromolecular scattering profiles are typically averaged and background subtracted, then basic analyses, such as a Guinier fit and calculation of molecular weight, are carried out. Except for *FIT2D*, *pyFAI* and *DPDAK*, all of the software listed above has these capabilities. Other common software for these operations are *ScÅtter*, *Primus* and the *ATSAS* utilities (Rambo, 2017 ▸; Konarev *et al.*, 2003 ▸; Petoukhov *et al.*, 2007 ▸, 2012 ▸; Franke *et al.*, 2017 ▸). After subtraction and basic quality checks, the choices for further analysis depend on the question(s) being investigated by the scientist. Many of the programs already mentioned have advanced analysis capabilities, as do others such as *GENFIT*, the *Integrative Modelling Platform* (*IMP*) SAXS software, *SASSIE*, *DENFERT*, *SASTBX*, *MEMPROT* (Curtis *et al.*, 2012 ▸; Liu *et al.*, 2012 ▸; Koutsioubas & Pérez, 2013 ▸; Spinozzi *et al.*, 2014 ▸; Pérez & Koutsioubas, 2015 ▸; Koutsioubas *et al.*, 2016 ▸; Perkins *et al.*, 2016 ▸; Schneidman-Duhovny *et al.*, 2016 ▸) and more (see, for example, http://smallangle.org/content/software). There are also programs specifically for analysis of size exclusion chromatography coupled SAXS (SEC-SAXS) data: *DATASW*, *DELA* and the US-SOMO HPLC-SAXS module (Malaby *et al.*, 2015 ▸; Shkumatov & Strelkov, 2015 ▸; Brookes *et al.*, 2016 ▸).

*RAW* is unique in that it (i) calibrates, masks and integrates images, (ii) can carry out basic data processing such as averaging, subtraction, Guinier fits and molecular weight calculation, (iii) incorporates advanced processing such as calculation of inverse Fourier transforms and envelopes, (iv) provides basic and advanced SEC-SAXS analysis capabilities, (v) can be used both at a beamline and by scientists on their personal machines, (vi) is open source, (vii) is free to all, and (viii) is available on all major OS platforms. The closest comparison among currently available software is to *Primus* and *ScÅtter*, both of which have features (ii), (iii), (v) and (viii), while *ScÅtter* additionally has feature (vi). Each of the three programs, *Primus*, *ScÅtter* and *RAW*, has features that are unique. The synchrotron beamline pipelines mentioned above also provide many of these features [at least (i)–(iii)], but cannot easily be used on scientists’ personal machines or at other beamlines. *RAW* is ideal for use both at a beamline and by scientists at home for initial data processing, validation and modeling. For these reasons, *RAW* is already used at several beamlines around the world (Acerbo *et al.*, 2015 ▸; Li *et al.*, 2016 ▸) and at numerous home sources.

This paper describes the new or updated features of *RAW* since the initial publication about the software (Nielsen *et al.*, 2009 ▸). These new and updated features include (i) improvements to the automatic centering and calibration routines, (ii) support for new detector types and new integrated (one-dimensional) data formats such as .dat [the standard extension for ASCII-formatted SAXS files with three-column data of *q*, *I*(*q*) and Δ*I*(*q*)], (iii) calculation of Guinier fits manually and automatically, (iv) calculation of molecular weight *via* four different methods, (v) control of *GNOM*, *AMBIMETER*, *DAMMIF*, *DAMAVER* and *DAMCLUST* (Svergun, 1992 ▸; Volkov & Svergun, 2003 ▸; Franke & Svergun, 2009 ▸; Petoukhov *et al.*, 2012 ▸; Petoukhov & Svergun, 2015 ▸; Franke *et al.*, 2017 ▸) from *RAW*, (vi) loading of SEC-SAXS data sets as intensity *versus* frame number curves and calculation of radius of gyration (*R*_g_) and molecular weight across peaks, (vii) singular value decomposition for analyzing the number of significant components in a data set, (viii) evolving factor analysis for model-free deconvolution of data based on the work of Meisburger *et al.* (2016 ▸), (ix) tracking of processing history and analysis, (x) availability on all major operating systems (Windows, Mac OSX, and Linux), and (xi) availability of a written tutorial and manual, and tutorial videos online.

## 2. Program overview   

*RAW* is a graphical user interface (GUI)-based free open-source program for reducing and analyzing biological small-angle X-ray scattering data. It is a Python-based program released under the GNU General Public License (GPL), with a few routines written in C++ for speed. Pre-built installers, source code, installation instructions, tutorials, manuals, version change logs and support are all available from the project web site: https://sourceforge.net/projects/bioxtasraw/. It has been designed as a quick to learn, easy to use program that allows users to process data from images to envelopes, and has been improved on the basis of feedback obtained from users at beamlines and in their home laboratories over the course of ∼8 years. It contains significant standalone data reduction and analysis features while also serving as a front end for some existing analysis software from the *ATSAS* (Franke *et al.*, 2017 ▸) package (which is closed source, and is free for academic but not industrial users). This article describes version 1.2.1 of *RAW* only; features may differ in other versions.

## 3. Working with images and scattering profiles   

### 3.1. Loading and processing images   

Typical SAXS image processing has been described in detail elsewhere (*e.g.* Pauw, 2013 ▸). In brief, it usually proceeds as follows: collect image; apply any necessary corrections to the image, for example dark current subtraction; mask image to remove artifacts, for example detector panel gaps and beamstop shadow; radially average image into a scattering profile of intensity *versus q* using known calibration values (beam center, sample–detector distance, X-ray energy), also called integration; apply any necessary corrections to the scattering profile, for example the solid angle correction; normalize the data by incident intensity or other equivalent value; and possibly apply additional normalization/correction factors, for example putting the data on an absolute scale. *RAW* supports all these operations.

*RAW* supports 27 different detector image formats using both the *FabIO* library (Knudsen *et al.*, 2013 ▸) and custom-written functions implemented independently from that library, including reading in the image header for use in processing. The *FabIO* library supports many major image formats including CBF, HDF5 and Pilatus Tiff, while *RAW* additionally supports MPA (multiwire), SAXSLab300, FReLoN, FLICAM, ILL SANS D11 and Medoptics image formats. It also calls the *pyFAI* library (Ashiotis *et al.*, 2015 ▸) to automatically calculate sample–detector distance and beam center position using a known calibrant. Masking and integration are carried out as previously described (Nielsen *et al.*, 2009 ▸).

Scattering profile normalization can now use general mathematical expressions referencing information in the image header or in an external header file. Many beamlines write separate header files containing information necessary for image processing, such as ion chamber or beamstop diode counts. The format of these files is beamline specific. Currently *RAW* can read in separate header files from CHESS beamlines G1 and F2, MaxLab beamlines I711 and I911-4, APS beamline 18-ID (BioCAT), and SSRF beamline BL19U2. New formats are added by writing a parse function that takes a filename as input and returns a dictionary of header values. Users can also set *RAW* to calculate and apply an absolute scale factor based on measured standards.

All of the image processing settings, as well as settings for advanced processing, can be saved as a configuration file and loaded before image processing. Once set, these parameters are automatically applied to any images loaded into *RAW*, either manually or in the online mode, allowing easy processing of large numbers of images. The Quick Reduce feature of *RAW* can be used to process images into scattering profiles without having to load them into *RAW*, further speeding up the process.

### 3.2. Basics of working with scattering profiles   

*RAW* can create scattering profiles from images and load profiles in .csv, .dat, .fit, .fir, .int, .rad and .sub format from many beamlines and programs. Scattering profiles created by *RAW* are saved as three-column (*q*, intensity and error in intensity) space-separated .dat files with a footer that contains analysis and processing information in JSON format. These saved files are compatible with other standard programs such as the *ATSAS* software and *ScÅtter*.

Scattering profiles loaded into *RAW* are plotted and can be easily manipulated. Users may select Lin–Lin, Lin–Log, Log–Lin, Log–Log, Kratky, Guinier or Porod plot axes. Scattering profiles can be averaged, subtracted, merged, rebinned, interpolated, scaled, offset, and truncated at both low and high *q*. The main scattering profile manipulation interface and plot are shown in Fig. S1 of the supporting information. All of these features are accessible in the manipulation panel, through right-click context menus, and/or through the View and Tools menus in the top menu bar.

### 3.3. Guinier fit   

Users can carry out a Guinier fit on a scattering profile, fitting a straight line to 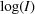 *versus* 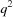 data, using the Guinier fit window (Fig. S2). The window displays the radius of gyration (*R*_g_) and scattering intensity at zero angle [*I*(0)] obtained from the fit, along with 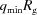, 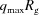 and the 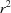 value of the fit. When this window is initially opened, *RAW* automatically attempts to find an optimal fit range. It does this using a heuristic approach that starts by selecting a set of ten windows of different sizes, with a minimum size of ten data points and a maximum size spanning from the minimum *q* value of the data set, *q*_min_, to the first *q* value where *I*(*q*_min_)/*I*(*q*) ≥ 10. Starting at the first point, these windows are stepped along the curve, with a step size equal to the number of *q* points divided by 50, and a Guinier fit is calculated for each interval (window plus start position). Each interval is assigned a quality score based on (i) how close 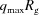 is to 1.3, (ii) how much less than 1 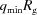 is, (iii) the size of the fractional errors in *R*_g_ and *I*(0), (iv) the 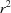 score of the fit, and (v) the length of the window (longer is better). More weight is given to (iv) and (v) than to the other parameters. A total score is assigned between 0 (minimum) and 1 (maximum). The window with the highest score, assuming any score over 0.6, is selected as the Guinier fit.

In addition to the automatic calculation, users may manually fit the curve, adjusting the start and end *q* points of the fit while watching the change in the plotted fit and the residual. This allows fine-tuning and evaluation of data quality problems or artifacts, particularly at low *q* where aggregation is most apparent. The fit data are saved with the scattering profile and can also be exported to a spreadsheet along with further analysis from the same and other scattering profiles. The analysis is saved when the scattering profile is saved, and can be viewed either in *RAW* or in a text editor. This is true for all analysis done on scattering profiles in *RAW*.

### 3.4. Molecular weight   

**Figure 1:**
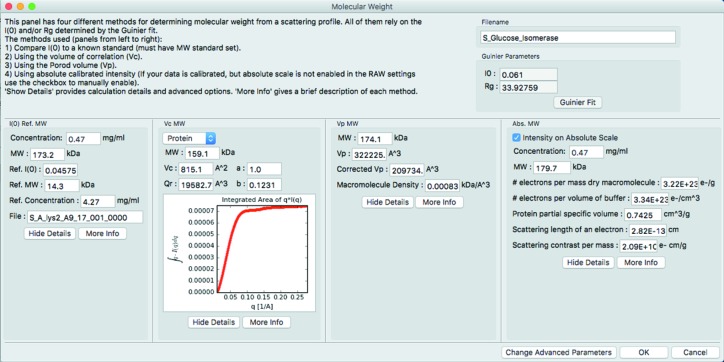
The molecular weight window, which can give up to four different estimates of molecular weight.

*RAW* has a molecular weight window (Fig. 1 ▸) which can calculate molecular weight (MW) by four methods: (i) reference of the *I*(0) value to that of a known standard, (ii) the absolute intensity value of *I*(0) (Orthaber *et al.*, 2000 ▸; Mylonas & Svergun, 2007 ▸), (iii) the adjusted Porod volume method (Fischer *et al.*, 2010 ▸) and (iv) the volume of correlation method (Rambo & Tainer, 2013 ▸). §S1 in the supporting information describes the validation of the implementations of methods (iii) and (iv) [for additional literature related to the supporting information see Valentini *et al.* (2015 ▸)]. Methods (i) and (ii) depend on an accurate measurement of the sample concentration, while methods (iii) and (iv) do not. Having multiple methods is important, as molecular weight determination from SAXS profiles is typically only accurate to ∼10% (Mylonas & Svergun, 2007 ▸), and different sources of error will affect the different methods.

In the original description of method (iii), the regularized intensity from an inverse Fourier transform (IFT) was used for the scattering profile, including extrapolation to *q* = 0. The implementation of method (iii) in *RAW* uses the Guinier fit to extrapolate to *q* = 0, so an IFT is not required. The regularized profile from an IFT can still be used to calculate MW by loading the IFT into *RAW*, sending the associated scattering profiles to the main plot and carrying out the analysis from there. Use of a Guinier extrapolation has independently been implemented in the *SAXS MoW2* calculator by the original authors of this method (http://saxs.ifsc.usp.br/).

### 3.5. IFT determination   

**Figure 2:**
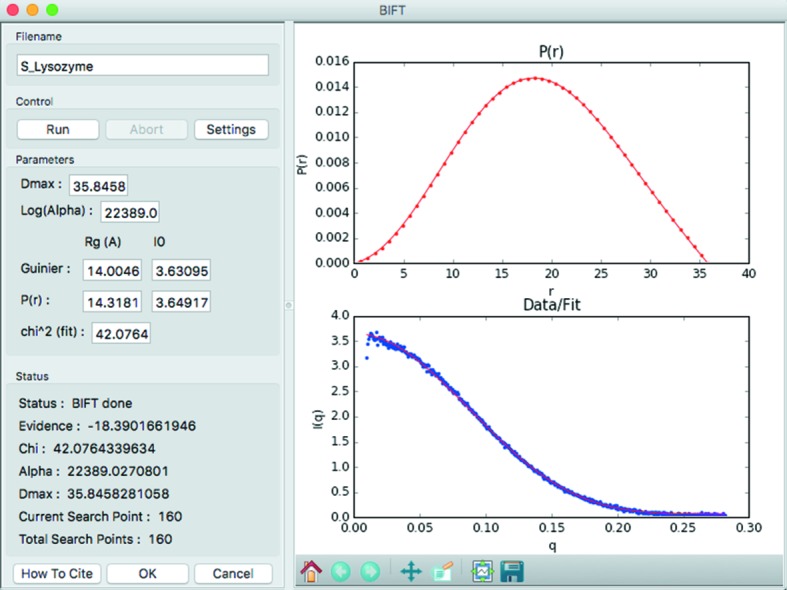
The BIFT window for determining IFT by that method.

Finding the IFT of a scattering profile in *RAW* can be done using two methods: a Bayesian method (Hansen, 2000 ▸), the implementation of which has been previously described (Nielsen *et al.*, 2009 ▸) (BIFT), and a method using the regularization parameter determined by perceptual criteria implemented in *GNOM* (Svergun, 1992 ▸). Figs. 2 ▸ and S3 show the new BIFT and GNOM windows, respectively. The GNOM window acts as a GUI for *GNOM* and *DATGNOM* from the *ATSAS* package (Petoukhov *et al.*, 2007 ▸), requiring a separate installation of *ATSAS* to work. The BIFT method is completely automatic once initial search parameters are set, while the GNOM window runs *DATGNOM* when first opened, and then allows manual adjustment of the maximum dimension (*D*_max_) and *q* range used. The advanced settings available for *GNOM* can be adjusted using the Advanced Settings GNOM panel. In both windows, the plots show the *P*(*r*) function and the data and regularized scattering profile. In the GNOM window the plots and results [such as *R*_g_ and *I*(0)] are updated as the user adjusts the controls. Once an IFT is determined, the user closes the window and the IFT is plotted in the IFT plot and control panels discussed in §4.

### 3.6. Manipulation history   

*RAW* tracks how a scattering profile was generated and what changes were made to the profile. For any scattering profile generated from an image, *RAW* tracks the configuration file used, the normalization parameters and the corrections (such as the solid-angle correction) used when integrating the image. For any scattering profile generated from other scattering profiles (such as an average profile), *RAW* keeps track of the scattering profiles used in the operation. For example, an average profile generated from ten individual profiles will have a history that lists the ten profiles used in the average. This history is recursively generated, so a subtracted profile generated from two averaged profiles will show the two profiles involved in the subtraction and the profiles involved in the average for each of those profiles. History is tracked for averaging, subtracting, merging, interpolation and binning. This history is visible from inside *RAW* and is saved as part of the footer in the .dat file as previously described. Once saved it can also be viewed in a text editor.

### 3.7. Exporting analysis information   

Analysis information, including *R*_g_, *I*(0), *D*_max_ and mol­ecular weight, may be exported from *RAW* scattering profiles as a comma-separated value (CSV) file. This allows users to easily track processing and compare results from different experiments. *RAW* can also export file header information and selectively export analysis information as a CSV file. The CSV file type was chosen as it can be directly read into common spreadsheet programs such as Microsoft *Excel* and *LibreOffice Calc* (The Document Foundation).

### 3.8. Online mode   

In online mode, *RAW* monitors a folder and loads in new files that are written in the folder. The basics of online mode were discussed previously (Nielsen *et al.*, 2009 ▸). There are new features that allow custom filtering of what files are loaded and detect modified files to reload when appropriate.

## 4. Working with IFTs   

### 4.1. Loading and plotting IFT data   

*RAW* allows users to load IFT files generated by *RAW* or *GNOM* and plot them in the IFT window (Fig. S4). This window is also where IFTs generated within *RAW*, using either BIFT or *GNOM* as in §3.5, are plotted. This allows comparison of *P*(*r*) functions, and the experimental data and regularized intensity can be loaded as scattering profiles in the main plot using the right-click menu. IFTs can be saved: those generated by BIFT are saved as .ift files, while those generated by *GNOM* are saved in the standard .out file format compatible with other programs that require that input, such as many programs in the *ATSAS* package.

### 4.2. Ambiguity determination   

Determination of envelopes from measured scattering is not unique, resulting in ambiguity in the final shape (Volkov & Svergun, 2003 ▸). *AMBIMETER* is a program that quantifies the ambiguity of shape determination from IFT data (Petoukhov & Svergun, 2015 ▸). It is available as a utility from the *ATSAS* package, and *RAW* implements a GUI for it (Fig. S5). The window reports the number of matching shapes, the ambiguity score and *AMBIMETER*’s assessment of the likelihood of a unique shape reconstruction. It also allows the user to save either the best-fit shape or all shapes from the *AMBIMETER* library that match the scattering profile. This requires a separate installation of the *ATSAS* package.

### 4.3. Envelope reconstruction   

**Figure 3:**
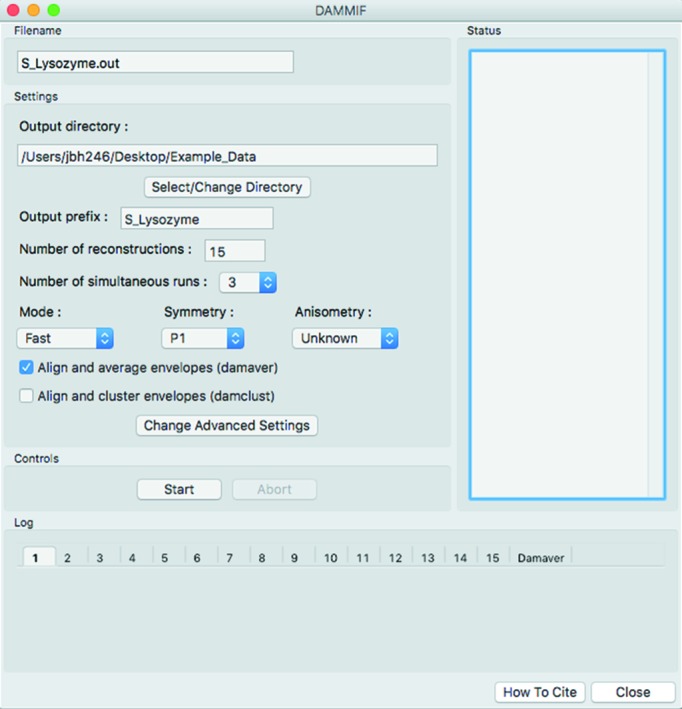
The DAMMIF window, which allows users to generate envelopes from IFT curves using the *ATSAS* program *DAMMIF*.

*RAW* allows users to generate envelopes from IFT data using the DAMMIF window (Fig. 3 ▸), which provides a GUI interface for the *DAMMIF*, *DAMAVER* and *DAMCLUST* programs of the *ATSAS* package (Volkov & Svergun, 2003 ▸; Franke & Svergun, 2009 ▸; Petoukhov *et al.*, 2012 ▸; Franke *et al.*, 2017 ▸). *RAW* will start a number of simultaneous *DAMMIF* runs set by the user (to a maximum equal to the number of processors or requested reconstructions, whichever is less). New runs are automatically started when runs finish, until all requested reconstructions have been made. Users may choose to then automatically run *DAMAVER* or *DAMCLUST* on the output. Users control common settings from the DAMMIF window, while advanced parameters can be set in the Advanced Settings DAMMIF window. As with the other *ATSAS* tools, use of *DAMMIF*, *DAMAVER* and *DAMCLUST* requires a separate *ATSAS* installation. The *DAMMIF* control runs in a separate thread from the main *RAW* program, so users can carry out other processing while waiting for reconstructions to finish.

## 5. Working with SEC data   

### 5.1. Loading and plotting liquid chromatography data   

Liquid chromatography coupled SAXS (LC-SAXS) data are data collected while the output of a fast protein/high-performance liquid chromatography (FPLC/HPLC) column flows through a SAXS sample cell (Mathew *et al.*, 2004 ▸). Typically a sizing column is used to separate the desired sample from aggregates or other contaminants, ensuring monodisperse data collection, and this is called size exclusion chromatography coupled SAXS (SEC-SAXS). Other types of separation can be used; for example ion exchange chromatography coupled SAXS (IEC-SAXS) was recently demonstrated (Hutin *et al.*, 2016 ▸). For LC-SAXS, images are collected continuously while the column elutes and the eluate flows through the sample cell. A typical data set consists of initial buffer images, images from the sample in one or more elution peaks from the column, and buffer images once all of the sample has eluted. As an LC-SAXS data set may contain hundreds or thousands of images, researchers have found plotting image intensity (total intensity or intensity at a particular *q* value) *versus* image number a useful initial representation of the data (Brookes *et al.*, 2013 ▸, 2016 ▸; Graewert *et al.*, 2015 ▸; Malaby *et al.*, 2015 ▸; Shkumatov & Strelkov, 2015 ▸; Brennich *et al.*, 2016 ▸; Hutin *et al.*, 2016 ▸). As the protein scatters more strongly than the buffer, this plot is mostly analogous to a SEC UV chromatograph (plotted as time or elution volume *versus* absorbance).

**Figure 4:**
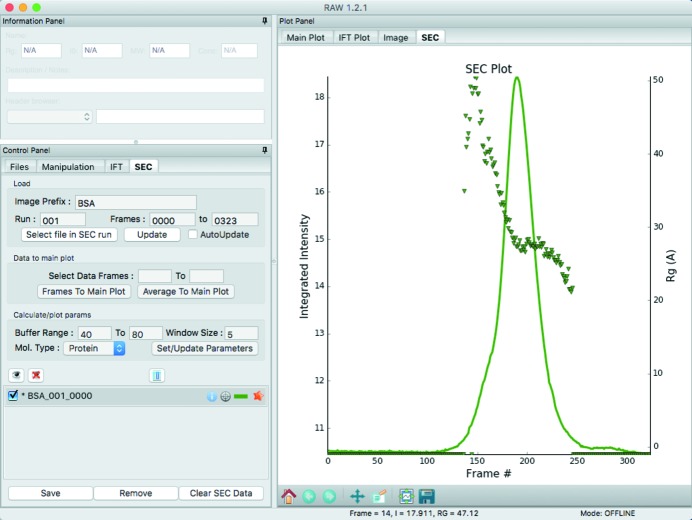
The SEC-SAXS control window and plot, showing intensity *versus* frame number (line) and *R*_g_ *versus* frame number (points) for BSA. The shoulder in the intensity and the increase in *R*_g_ show that the monomer and oligomer did not fully separate.

In *RAW*, users may load any set of scattering profiles as a ‘SEC’ curve. This allows users to plot total intensity, average intensity or intensity at any arbitrary *q* value *versus* ‘frame number’ (the index of the data item relative to the first data item), as shown in Fig. 4 ▸. *RAW* also has automated loading for SEC-SAXS data from beamlines where the file naming convention is known. At these beamlines, SEC-SAXS data can be loaded in an online mode, where the intensity *versus* frame number curve is updated whenever new frames are collected. Because of the open-source nature of *RAW*, anyone could add a new file naming convention and enable online mode for SEC-SAXS data for another beamline.

### 5.2. Calculating *R*_g_, molecular weight and *I*(0) across SEC peaks   

After plotting intensity *versus* frame number, the usual next step in SEC-SAXS data processing is to calculate structural parameters as a function of frame number, allowing the scientist to assess both content and quality of the data (Brookes *et al.*, 2013 ▸, 2016 ▸; Graewert *et al.*, 2015 ▸; Malaby *et al.*, 2015 ▸; Shkumatov & Strelkov, 2015 ▸; Brennich *et al.*, 2016 ▸; Hutin *et al.*, 2016 ▸). *RAW* allows the user to define a set of frames as the buffer range and the number of scattering profiles to average together to calculate structural parameters. All the scattering profiles in the defined buffer range are averaged and this average is subtracted from all of the scattering profiles in the data set. A window of the defined average size is then stepped along the data, all scattering profiles within it are averaged, and an attempt is made to automatically calculate *R*_g_, *I*(0) and the molecular weight. In this instance, *RAW* uses the automatic *R*_g_ determination function described earlier and the volume of correlation method (the volume of correlation method does not require sample concentration and can handle flexible proteins and RNA with default settings). For example, if the user sets a five-frame average size, the buffer-subtracted scattering profiles from frames 1–5, 2–6, 3–7 *etc*. would be averaged and structural parameters calculated for each of those averages. The user can choose to plot *R*_g_, molecular weight or *I*(0) *versus* frame number on the same plot as the intensity *versus* frame number, as shown in Fig. 4 ▸. If online mode is enabled and the user has defined the buffer range and average window size, these calculations are carried out on each new frame as it is loaded into *RAW*.

### 5.3. Extracting, saving and exporting data for further analysis   

From SEC data in *RAW* users can extract individual scattering profiles of interest, save the SEC curve for further analysis in *RAW* or export data about the SEC curve for use in any other program. To extract individual scattering profiles, users select a range of frame numbers and send either each scattering profile or the average scattering profile of the selected frames to the main plot. Users can save the entire SEC curve as a *RAW*-specific .sec file, making it simple to load every profile, subtracted profile, and calculated *R*_g_, molecular weight and *I*(0) value back into *RAW* to continue analysis at a later point. Additionally, users can export all of the following data as a CSV file: total intensity, average intensity, intensity at a particular *q*, frame number, *R*_g_, uncertainty in *R*_g_, molecular weight, *I*(0), uncertainty in *I*(0) and filename associated with each frame number. Finally, users can export all of the frame data as scattering profiles, which can be useful if images were loaded and the users wishes to save all of the scattering profiles *RAW* created from those images.

## 6. Singular value decomposition and evolving factor analysis   

### 6.1. Singular value analysis   

Singular value decomposition (SVD) is a mathematical technique that provides model-independent information on the number of unique elements in a data set. SVD has frequently been used to analyze mixture and time-resolved SAXS data (Doniach, 2001 ▸; Svergun & Koch, 2003 ▸; Petoukhov & Svergun, 2007 ▸; Mertens & Svergun, 2010 ▸; Pollack, 2011 ▸; Schneidman-Duhovny *et al.*, 2012 ▸; Blanchet & Svergun, 2013 ▸). Formally, singular value decomposition of an *m* × *n* matrix *M* is a factorization into three matrices such that 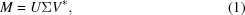
where *U* is an 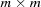 unitary matrix, called the left singular vectors; Σ is a diagonal *m* × *n* matrix, where the diagonal values are the singular values; and V* is the conjugate transpose of an *n* × *n* unitary matrix *V*, the right singular vectors. A typical interpretation of SVD is that the number of singular values significantly above the baseline level is the number of independent components in the data set.

**Figure 5:**
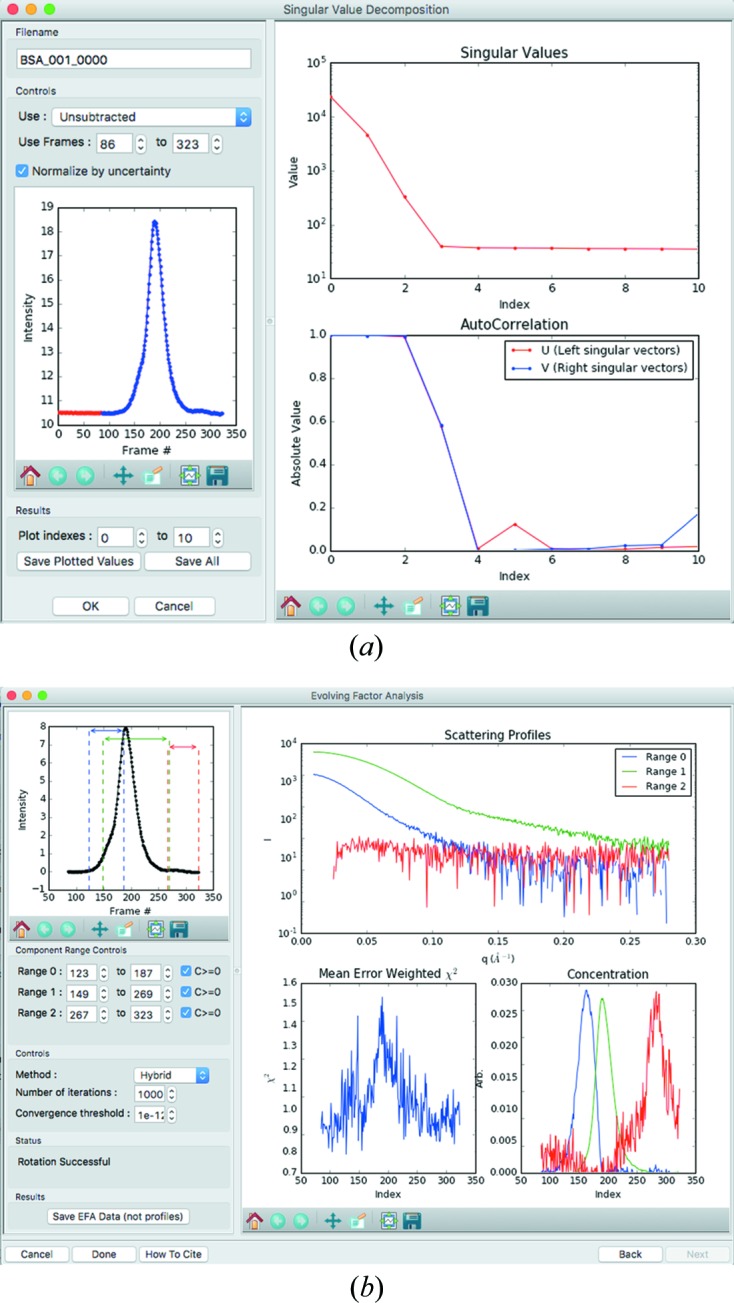
(*a*) The SVD analysis window, showing the selected data range (blue points on the left) and plots of the first ten singular values and left and right singular vector autocorrelations. This shows three significant singular values in the data set, attributable to the scattering from the buffer, BSA monomer and BSA oligomer. (*b*) The final window of EFA in *RAW*, showing the selected data ranges, the extracted scattering profiles from the overlapping peaks and other information.

*RAW* can perform SVD on scattering profiles or *P*(*r*) functions. This is typically applied to scattering profiles in a SEC-SAXS data set, and the number of significant singular values corresponds to the number of distinct scatterers in the data set. SVD done on a single well separated peak from the chromatograph would yield two significant components: one from the buffer and one from the macromolecule. For SVD done on a poorly separated monomer–dimer peak there would be three significant components: buffer, monomer and dimer. *RAW* allows users to select a range of scattering profiles for SVD and displays the singular values, σ_*i*_, and the autocorrelation of the left and right singular vectors, *R_i_*, for each *i*th singular value, defined as 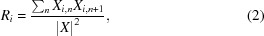
where *X* is the *U* or *V* singular vector matrix. Autocorrelation values and the magnitude of the singular value allow the user to interpret which singular values are significant. Fig. 5 ▸(*a*) shows the SVD analysis window.

### 6.2. Evolving factor analysis   

Evolving factor analysis (EFA) is an extension of SVD to allow model-independent separation of scattering profiles from mixed solutions, particularly overlapping chromatographic peaks (Maeder, 1987 ▸; Maeder & Neuhold, 2007 ▸). This method was recently applied to SEC-SAXS data (Meisburger *et al.*, 2016 ▸), and an improved version of the method described by Meisburger *et al.* has been implemented in *RAW*. EFA in *RAW* starts with SVD, proceeds by finding the component start and end points in the evolving factor plots, and finally rotates the significant singular value vectors into scattering profiles. *RAW* implements two new methods for rotation of the singular vectors besides the iterative approach of Meisburger *et al.* (2016 ▸). The first is the explicit calculation method described by Maeder (1987 ▸). The second is a hybrid method that uses the explicit calculation as the seed for the iterative approach, giving faster convergence of the rotation. Fig. 5 ▸(*b*) shows the final window of the EFA analysis in *RAW*, with extracted scattering profiles from overlapping peaks. EFA is different from other common deconvolution techniques, such as those implemented in *US-SOMO* and *DELA* (Brookes *et al.*, 2013 ▸, 2016 ▸; Malaby *et al.*, 2015 ▸), in that it is a model-free approach. However, it is not without its own limitations. §S2 contains the mathematics of our EFA approaches, some remarks on validation of EFA compared with the original instance and our experience with practical limitations to the current implementation of the technique.

## 7. Conclusions and future outlook   

*RAW* is a free open-source program for calibrating, masking, integrating and analyzing biological SAXS data. It provides the ability to analyze standard SAXS data and SEC-SAXS data, including Guinier analysis, molecular weight calculation, calculation of *P*(*r*) functions, singular value decomposition and evolving factor analysis, and the use of the *AMBIMETER*, *DAMMIF*, *DAMAVER*, *DAMCLUST*, *DATGNOM* and *GNOM* programs from the *ATSAS* package (which is closed source, requires a separate installation, and is free for academic but not industrial users). *RAW* is available from https://sourceforge.net/projects/bioxtasraw/. It is written in the Python and C++ programming languages and runs on all major operating systems. It has detailed documentation and video tutorials available online.

Development, including new features, speed improvements and bug fixes, is ongoing, and other scientists are welcome to contribute to *RAW* development. In the future we intend to add major features including, but not limited to, normalized Kratky plots, a multi-detector mode to seamlessly generate scattering profiles from measurements on several detectors, improved azimuthal integration with support for detector tilt parameters, flexibility analysis, some level of automated processing of subtracted profiles and similarity testing for scattering profiles.

Looking forward, data processing pipelines are becoming more popular at dedicated biological small-angle scattering beamlines. However, not all analysis can be done at the beamline, and scientists will continue to require portable analysis software that they can run at home. Furthermore, many biological experiments are done at small-angle scattering beamlines or home sources not dedicated to biological samples, and these experiments will require some kind of standalone analysis program. Thus, at least for the foreseeable future, we anticipate that *RAW* and programs like *RAW* will continue to be useful for the community.

## 8. Note added in proof   

During review and publication of this paper several new versions of *RAW* were released, adding some significant new features. As of publication, the newest version is 1.3.0, and includes the following major features not described in this paper: ability to use *DAMMIN* (Svergun, 1999 ▸) as well as *DAMMIF* for envelope reconstructions and to refine averaged reconstructions; a summary window for envelope reconstructions which shows the chi squared, radius of gyration, maximum dimension, excluded volume and estimated mol­ecular weight for each reconstruction, as well as (when available) the normalized spatial discrepancy, number of models included in the average, clustering of models, ambiguity of the reconstruction and resolution of the reconstruction; similarity testing of profiles using the CorMap test (Franke *et al.*, 2015 ▸), available as a tool for the user and carried out automatically when averaging profiles; absolute scaling using glassy carbon (Zhang *et al.*, 2010 ▸; Allen *et al.*, 2017 ▸); and normalized and dimensionless Kratky plotting (Durand *et al.*, 2010 ▸; Rambo & Tainer, 2011 ▸; Receveur-Bréchot & Durand, 2012 ▸).

## Supplementary Material

Supporting information. DOI: 10.1107/S1600576717011438/ge5036sup1.pdf

## Appendix A Python libraries used

*RAW* currently requires Python 2.7 to run. It also uses a number of third-party Python packages. These include the *Numpy* (Van Der Walt *et al.*, 2011 ▸), *Matplotlib* (Hunter, 2007 ▸), *Scipy* (Jones *et al.*, 2001 ▸), *h5py* (Collette, 2008 ▸), *FabIO* (Knudsen *et al.*, 2013 ▸), *pyFAI* (Ashiotis *et al.*, 2015 ▸), *Pillow* (https://python-pillow.org/), *wxPython* (https://wxpython.org/), *lxml* (http://lxml.de/) and *hdf5plugin* (https://github.com/silx-kit/hdf5plugin) packages and all of their dependencies. All of these packages, their dependencies and Python (version >2.0) are licensed with GPL compatible licenses.

## References

[bb1] Acerbo, A. S., Cook, M. J. & Gillilan, R. E. (2015). *J. Synchrotron Rad.* **22**, 180–186. 10.1107/S1600577514020360 PMC4294029 25537607

[bb2] Allen, A. J., Zhang, F., Kline, R. J., Guthrie, W. F. & Ilavsky, J. (2017). *J. Appl. Cryst.* **50**, 462–474. 10.1107/S1600576717001972 PMC5377342 28381972

[bb3] Ashiotis, G., Deschildre, A., Nawaz, Z., Wright, J. P., Karkoulis, D., Picca, F. E. & Kieffer, J. (2015). *J. Appl. Cryst.* **48**, 510–519. 10.1107/S1600576715004306 PMC4379438 25844080

[bb4] Benecke, G., Wagermaier, W., Li, C., Schwartzkopf, M., Flucke, G., Hoerth, R., Zizak, I., Burghammer, M., Metwalli, E., Müller-Buschbaum, P., Trebbin, M., Förster, S., Paris, O., Roth, S. V. & Fratzl, P. (2014). *J. Appl. Cryst.* **47**, 1797–1803. 10.1107/S1600576714019773 PMC4180741 25294982

[bb5] Blanchet, C. E., Spilotros, A., Schwemmer, F., Graewert, M. A., Kikhney, A., Jeffries, C. M., Franke, D., Mark, D., Zengerle, R., Cipriani, F., Fiedler, S., Roessle, M. & Svergun, D. I. (2015). *J. Appl. Cryst.* **48**, 431–443. 10.1107/S160057671500254X PMC4379436 25844078

[bb6] Blanchet, C. E. & Svergun, D. I. (2013). *Annu. Rev. Phys. Chem.* **64**, 37–54. 10.1146/annurev-physchem-040412-110132 23216378

[bb7] Brennich, M. E., Kieffer, J., Bonamis, G., De Maria Antolinos, A., Hutin, S., Pernot, P. & Round, A. (2016). *J. Appl. Cryst.* **49**, 203–212.

[bb8] Brookes, E., Pérez, J., Cardinali, B., Profumo, A., Vachette, P. & Rocco, M. (2013). *J. Appl. Cryst.* **46**, 1823–1833. 10.1107/S0021889813027751 PMC3831300 24282333

[bb9] Brookes, E., Vachette, P., Rocco, M. & Pérez, J. (2016). *J. Appl. Cryst.* **49**, 1827–1841. 10.1107/S1600576716011201 PMC5045733 27738419

[bb10] Chaudhuri, B. N. (2015). *Protein Sci.* **24**, 267–276. 10.1002/pro.2624 PMC4353354 25516491

[bb11] Collette, A. (2008). *HDF5 for Python*, http://www.h5py.org/.

[bb12] Curtis, J. E., Raghunandan, S., Nanda, H. & Krueger, S. (2012). *Comput. Phys. Commun.* **183**, 382–389.

[bb13] Doniach, S. (2001). *Chem. Rev.* **101**, 1763–1778. 10.1021/cr990071k 11709998

[bb14] Durand, D., Vivès, C., Cannella, D., Pérez, J., Pebay-Peyroula, E., Vachette, P. & Fieschi, F. (2010). *J. Struct. Biol.* **169**, 45–53. 10.1016/j.jsb.2009.08.009 19723583

[bb15] Dyer, K. N., Hammel, M., Rambo, R. P., Tsutakawa, S. E., Rodic, I., Classen, S., Tainer, J. A. & Hura, G. L. (2014). *Structural Genomics: General Applications*, edited by Y. W. Chen, pp. 245–258. Totowa: Humana Press.

[bb16] Fischer, H., de Oliveira Neto, M., Napolitano, H. B., Polikarpov, I. & Craievich, A. F. (2010). *J. Appl. Cryst.* **43**, 101–109.

[bb17] Franke, D., Jeffries, C. M. & Svergun, D. I. (2015). *Nat. Methods*, **12**, 419–422. 10.1038/nmeth.3358 25849637

[bb18] Franke, D., Kikhney, A. G. & Svergun, D. I. (2012). *Nucl. Instrum. Methods Phys. Res. Sect. A*, **689**, 52–59.

[bb68] Franke, D., Petoukhov, M. V., Konarev, P. V., Panjkovich, A., Tuukkanen, A., Mertens, H. D. T., Kikhney, A. G., Hajizadeh, N. R., Franklin, J. M., Jeffries, C. M. & Svergun, D. I. (2017). *J. Appl. Cryst.* **50**, 1212–1225. 10.1107/S1600576717007786 PMC5541357 28808438

[bb19] Franke, D. & Svergun, D. I. (2009). *J. Appl. Cryst.* **42**, 342–346. 10.1107/S0021889809000338 PMC5023043 27630371

[bb20] Graewert, M. A., Franke, D., Jeffries, C. M., Blanchet, C. E., Ruskule, D., Kuhle, K., Flieger, A., Schäfer, B., Tartsch, B., Meijers, R. & Svergun, D. I. (2015). *Sci. Rep.* **5**, 10734. 10.1038/srep10734 PMC5377070 26030009

[bb21] Graewert, M. A. & Svergun, D. I. (2013). *Curr. Opin. Struct. Biol.* **23**, 748–754. 10.1016/j.sbi.2013.06.007 23835228

[bb69] Hammersley, A. P. (2016). *J. Appl. Cryst.* **49**, 646–652.

[bb22] Hammersley, A. P., Svensson, S. O., Hanfland, M., Fitch, A. N. & Hausermann, D. (1996). *High. Pressure Res.* **14**, 235–248.

[bb23] Hansen, S. (2000). *J. Appl. Cryst.* **33**, 1415–1421.

[bb24] Hunter, J. D. (2007). *Comput. Sci. Eng.* **9**, 90–95.

[bb25] Hutin, S., Brennich, M., Maillot, B. & Round, A. (2016). *Acta Cryst.* D**72**, 1090–1099. 10.1107/S2059798316012833 PMC5053136 27710930

[bb26] Jacques, D. A. & Trewhella, J. (2010). *Protein Sci.* **19**, 642–657. 10.1002/pro.351 PMC2867006 20120026

[bb27] Jones, E., Oliphant, T., Peterson, P. *et al.* (2001). *Scipy: Open Source Scientific Tools for Python*, https://www.scipy.org/.

[bb28] Knudsen, E. B., Sørensen, H. O., Wright, J. P., Goret, G. & Kieffer, J. (2013). *J. Appl. Cryst.* **46**, 537–539.

[bb29] Konarev, P. V., Volkov, V. V., Sokolova, A. V., Koch, M. H. J. & Svergun, D. I. (2003). *J. Appl. Cryst.* **36**, 1277–1282.

[bb71] Koutsioubas, A., Jaksch, S. & Pérez, J. (2016). *J. Appl. Cryst.* **49**, 690–695.

[bb30] Koutsioubas, A. & Pérez, J. (2013). *J. Appl. Cryst.* **46**, 1884–1888.

[bb31] Li, N., Li, X., Wang, Y., Liu, G., Zhou, P., Wu, H., Hong, C., Bian, F. & Zhang, R. (2016). *J. Appl. Cryst.* **49**, 1428–1432. 10.1107/S160057671601195X PMC5045727 27738413

[bb32] Liu, H., Hexemer, A. & Zwart, P. H. (2012). *J. Appl. Cryst.* **45**, 587–593.

[bb33] Maeder, M. (1987). *Anal. Chem.* **59**, 527–530.

[bb34] Maeder, M. & Neuhold, Y.-M. (2007). *Practical Data Analysis in Chemistry*. Amsterdam: Elsevier.

[bb35] Malaby, A. W., Chakravarthy, S., Irving, T. C., Kathuria, S. V., Bilsel, O. & Lambright, D. G. (2015). *J. Appl. Cryst.* **48**, 1102–1113. 10.1107/S1600576715010420 PMC4520288 26306089

[bb36] Mathew, E., Mirza, A. & Menhart, N. (2004). *J. Synchrotron Rad.* **11**, 314–318. 10.1107/S0909049504014086 15211037

[bb70] McPhillips, T. M., McPhillips, S. E., Chiu, H.-J., Cohen, A. E., Deacon, A. M., Ellis, P. J., Garman, E., Gonzalez, A., Sauter, N. K., Phizackerley, R. P., Soltis, S. M. & Kuhn, P. (2002). *J. Synchrotron Rad.* **9**, 401–406. 10.1107/s0909049502015170 12409628

[bb37] Meisburger, S. P., Taylor, A. B., Khan, C. A., Zhang, S., Fitzpatrick, P. F. & Ando, N. (2016). *J. Am. Chem. Soc.* **138**, 6506–6516. 10.1021/jacs.6b01563 PMC4896396 27145334

[bb38] Mertens, H. D. T. & Svergun, D. I. (2010). *J. Struct. Biol.* **172**, 128–141. 10.1016/j.jsb.2010.06.012 20558299

[bb39] Mylonas, E. & Svergun, D. I. (2007). *J. Appl. Cryst.* **40**, s245–s249.

[bb40] Nielsen, S. S., Toft, K. N., Snakenborg, D., Jeppesen, M. G., Jacobsen, J. K., Vestergaard, B., Kutter, J. P. & Arleth, L. (2009). *J. Appl. Cryst.* **42**, 959–964.

[bb41] Orthaber, D., Bergmann, A. & Glatter, O. (2000). *J. Appl. Cryst.* **33**, 218–225.

[bb42] Pauw, B. R. (2013). *J. Phys. Condens. Matter*, **25**, 383201. 10.1088/0953-8984/25/38/383201 23988669

[bb43] Pérez, J. & Koutsioubas, A. (2015). *Acta Cryst.* D**71**, 86–93. 10.1107/S1399004714016678 PMC4304689 25615863

[bb44] Perkins, S. J., Wright, D. W., Zhang, H., Brookes, E. H., Chen, J., Irving, T. C., Krueger, S., Barlow, D. J., Edler, K. J., Scott, D. J., Terrill, N. J., King, S. M., Butler, P. D. & Curtis, J. E. (2016). *J. Appl. Cryst.* **49**, 1861–1875. 10.1107/S160057671601517X PMC5139988 27980506

[bb45] Petoukhov, M. V., Franke, D., Shkumatov, A. V., Tria, G., Kikhney, A. G., Gajda, M., Gorba, C., Mertens, H. D. T., Konarev, P. V. & Svergun, D. I. (2012). *J. Appl. Cryst.* **45**, 342–350. 10.1107/S0021889812007662 PMC4233345 25484842

[bb46] Petoukhov, M. V., Konarev, P. V., Kikhney, A. G. & Svergun, D. I. (2007). *J. Appl. Cryst.* **40**, s223–s228.

[bb47] Petoukhov, M. V. & Svergun, D. I. (2007). *Curr. Opin. Struct. Biol.* **17**, 562–571. 10.1016/j.sbi.2007.06.009 17714935

[bb48] Petoukhov, M. V. & Svergun, D. I. (2013). *Int. J. Biochem. Cell Biol.* **45**, 429–437. 10.1016/j.biocel.2012.10.017 23142499

[bb49] Petoukhov, M. V. & Svergun, D. I. (2015). *Acta Cryst.* D**71**, 1051–1058. 10.1107/S1399004715002576 25945570

[bb50] Pollack, L. (2011). *Biopolymers*, **95**, 543–549. 10.1002/bip.21604 21328311

[bb53] Rambo, R. P. (2017). *ScÅtter*, http://www.bioisis.net/tutorial/9.

[bb51] Rambo, R. P. & Tainer, J. A. (2011). *Biopolymers*, **95**, 559–571. 10.1002/bip.21638 PMC3103662 21509745

[bb52] Rambo, R. P. & Tainer, J. A. (2013). *Nature*, **496**, 477–481. 10.1038/nature12070 PMC3714217 23619693

[bb54] Receveur-Bréchot, V. & Durand, D. (2012). *Curr. Protein Pept. Sci.* **13**, 55–75. 10.2174/138920312799277901 PMC3394175 22044150

[bb55] Schneidman-Duhovny, D., Hammel, M., Tainer, J. A. & Sali, A. (2016). *Nucleic Acids Res.* **44**, W424–W429. 10.1093/nar/gkw389 PMC4987932 27151198

[bb56] Schneidman-Duhovny, D., Kim, S. J. & Sali, A. (2012). *BMC Struct. Biol.* **12**, 1–12. 10.1186/1472-6807-12-17 PMC3427135 22800408

[bb57] Shkumatov, A. V. & Strelkov, S. V. (2015). *Acta Cryst.* D**71**, 1347–1350. 10.1107/S1399004715007154 26057674

[bb58] Skou, S., Gillilan, R. E. & Ando, N. (2014). *Nat. Protoc.* **9**, 1727–1739. 10.1038/nprot.2014.116 PMC4472361 24967622

[bb59] Spinozzi, F., Ferrero, C., Ortore, M. G., De Maria Antolinos, A. & Mariani, P. (2014). *J. Appl. Cryst.* **47**, 1132–1139. 10.1107/S1600576714005147 PMC4038801 24904247

[bb60] Svergun, D. I. (1992). *J. Appl. Cryst.* **25**, 495–503.

[bb61] Svergun, D. I. (1999). *Biophys. J.* **76**, 2879–2886. 10.1016/S0006-3495(99)77443-6 PMC1300260 10354416

[bb62] Svergun, D. I. & Koch, M. H. J. (2003). *Rep. Prog. Phys.* **66**, 1735–1782.

[bb63] Valentini, E., Kikhney, A. G., Previtali, G., Jeffries, C. M. & Svergun, D. I. (2015). *Nucleic Acids Res.* **43**, D357–D363. 10.1093/nar/gku1047 PMC4383894 25352555

[bb64] Vestergaard, B. & Sayers, Z. (2014). *IUCrJ*, **1**, 523–529. 10.1107/S2052252514020843 PMC4224470 25485132

[bb65] Volkov, V. V. & Svergun, D. I. (2003). *J. Appl. Cryst.* **36**, 860–864.

[bb66] Walt, S. van der, Colbert, S. C. & Varoquaux, G. (2011). *Comput. Sci. Eng.* **13**, 22–30.

[bb67] Zhang, F., Ilavsky, J., Long, G. G., Quintana, J. P. G., Allen, A. J. & Jemian, P. R. (2010). *Metall. Mater. Trans. A*, **41**, 1151–1158.

