# Supplementary material for: BioXTAS RAW: improvements to a free open-source program for small-angle X-ray scattering data reduction and analysis
Source: J Appl Crystallogr. 2017 Sep 5;50(Pt 5):1545–53. doi: 10.1107/S1600576717011438 (PMC5627684; doi:10.1107/S1600576717011438)
Supplement: Supplementary file 1 [file j-50-01545-sup1.pdf]

JOURNAL OF
APPLIED
CRYSTALLOGRAPHY

Volume 50 (2017)

Supporting information for article:

BioXTAS RAW: improvements to a free open-source program for small-angle X-ray scattering data reduction and analysis

Jesse Bennett Hopkins, Richard E. Gillilan and Soren Skou

Supporting Information

S1. Validation of molecular weight methods

S1.1. Adjusted Porod volume method

In order to validate our implementation of the adjusted Porod volume method of (Fischer et al., 2010), we compared the molecular weight calculation from the SAXS MoW2 calculator (http://saxs.ifsc.usp.br/) with the results from RAW for several different proteins and q ranges. The protein scattering profiles were taken from the SASBDB (https://www.sasbdb.org/) (Valentini et al., 2015) and BIOSIS (http://www.bioisis.net/). These proteins were chosen to have a large total *q* range, a wide range of sizes, and so that some of the scattering profiles had imperfect Guinier regions (either due to aggregation, repulsion, or bad subtraction). The results are shown in Table S1, and show that within the *q*-ranges for correction given in the original paper, results from the two programs agree within 3% in all cases, and on average across all proteins and q ranges tested the results from the two programs agree to better than 0.01%. The largest discrepancy is seen for a protein that has obvious aggregation at low *q* (SASDA59), and so may result from differences in how the Guinier extrapolation is being done in the two programs.

S1.2. Volume of correlation method

In order to validate our implementation of the volume of correlation method of (Rambo & Tainer, 2013) we compared the volume of correlation molecular weight results from ScÅtter (version 3.0j, which was created by the original authors of the method) and RAW. We tested the same set of proteins used to test the Adjusted Porod volume method in Section S1.1. The results are shown in Table S2. ScÅtter does not report a MW for a maximum *q* value of 0.2 Å^{-1}, so only two of the maximum *q* values reported in Table S1 are given.

For a maximum *q* value 0.3 Å^{-1} the two implementations agree essentially identical, with an average discrepancy of 0.005% and a maximum discrepancy of 0.04%. There is significant disagreement for the maximum *q* value of 0.4, as, based on empirical testing, ScÅtter seems to only integrate the volume of correlation to a maximum of q~0.32 Å^{-1} regardless of scattering profile used. The reason for this truncation is not known. RAW integrates to the maximum *q* value of the provided profile (in this test case 0.4 Å^{-1}), hence the difference.

S2. Further remarks on evolving factor analysis

S2.1. Mathematics of EFA

Evolving Factor Analysis is well described in (Maeder, 1987; Maeder & Neuhold, 2007; Meisburger et al., 2016). Because it is a new technique for the SAXS field we describe it below, in the notation

style of (Meisburger et al., 2016). The three methods described in the main text are the iterative, hybrid, and explicit methods. These refer to three different ways of rotating the SVD basis vectors into the original basis (scattering profiles vs. elution volume). We provide an overview of the method first, and then the details of each rotation method.

S2.1.1. Overview of EFA

EFA starts with a set of SEC-SAXS scattering profiles of total number *m* all belonging to an elution series, with intensity $I_j(q)$ and associated experimental errors $\Delta I_j(q)$ where the subscript indicates the *j*-th profile in the elution series. Profiles are assumed to be loaded sequentially by elution volume, with the *j=1* profile corresponding to the earliest measured elution volume. Intensity and error matrices $\mathbf{D}$ and $\mathbf{E}$ are prepared where the column vectors are $I_j(q)$ and $\Delta I_j(q)$ respectively, such that $D_{ij} = I_j(q_i)$ and $E_{ij} = \Delta I_j(q_i)$. Finally, an error weighted scattering profile matrix, $\mathbf{A}$, is prepared,

$$A_{ij} = D_{ij} / \left(\frac{1}{m} \sum_{j=1}^{m} E_{ij} \right), \quad (1)$$

such that each *q*-bin contributes variance of ~1.

Singular value decomposition (SVD) of $\mathbf{A}$ is then carried out as

$$\mathbf{A} = \mathbf{USV}^T . \quad (2)$$

Once the SVD is carried out, users graphically identify the number of significant singular values in their data set, n_s. Forward and backward EFA are then done, to determine where the elution peaks begin/end. Forward EFA consists of SVD repeatedly carried out on a portion of the $\mathbf{A}$ matrix, $\mathbf{A}_l$, where $\mathbf{A}_l$ is defined as the first *l* columns of $\mathbf{A}$. Letting *l* vary from 1 to the *m*, the values of the first $n_s + 1$ singular values are plotted vs. *l*. This plot is the Forward Evolving Factor plot. Backward EFA is the same as forward EFA, but instead of taking the first *l* columns of $\mathbf{A}$, the last *l* columns of $\mathbf{A}$ are used. This is plotted on the Backward Evolving Factor plot. Users then select the points of the forward/backward evolving factors where each factor (singular value vs. profile number) starts to rise above the baseline. These points are represented by the vectors $\mathbf{p}^{(f)}$ and $\mathbf{p}^{(b)}$, which are sorted in ascending order, for the forward and backward points respectively.

It is assumed that components elute serially, so the first point in $\mathbf{p}^{(f)}$ and $\mathbf{p}^{(b)}$ represents the start and end respectively of the first component in the data. This assumption is used by all of the rotation methods described below to rotate from the SVD basis back to the original basis, which results in the following outputs: $\mathbf{C}$, a matrix containing the concentration profiles for each pure component, $J_k(q)$

the scattering profiles of each pure component, and $\Delta J_k(q)$ the errors for each pure component. Here, the subscript indicates the *k*-th pure component, where *k* is between 1 and n_s.

S2.1.2. Iterative rotation method

The iterative rotation method is that used by (Meisburger et al., 2016). This description generally follows the program laid out in their Figure S11. The first step is initialization. We start by making an initial concentration matrix $\mathbf{C}$ for the pure components. Note that $\mathbf{C}$ has n_s number of columns. If no previous successful rotation of the basis vectors has been achieved, then we set $\mathbf{C}$ equal to the first n_s columns of $\mathbf{V}$, $C_{jk} = V_{jk}$. Otherwise, we set $\mathbf{C}$ equal to the concentration matrix of the successful rotation (for example, this happens if the user has achieved a successful rotation, then changes the range of one or more of the components by changing the selected inflection point in $\mathbf{p}^{(f)}$ and/or $\mathbf{p}^{(b)}$). The use of the previous successful rotation as an initial value is purely to speed up convergence of the iterative algorithm.

Next, we create the indicator matrix $\mathbf{M}$, where each column represents the range of a component in the data, i.e. is 1 where the component exists in the data set and is zero otherwise:

$$M_{jk} = \begin{cases} 1 & \mathbf{p}_k^{(f)} \le j \le p_k^{(b)} \\ 0 & \text{otherwise} \end{cases} . \tag{3}$$

We define that the algorithm has converged when the absolute change in $\mathbf{C}$ is less than δ_0, a tolerance threshold selected by the user. The user also selects the maximum number of iterations at which the algorithm will terminate if convergence is not achieved. In RAW, reasonable default values of these parameters are provided.

Once the initialization is done, the algorithm enters the iterative stage. First, we compute the basis set from the Moore-Penrose pseudoinverse of the masked concentration matrix

$$\mathbf{G} = \mathbf{A}\left(\left(\mathbf{M} \circ \mathbf{C}\right)^T\right)^+ , \tag{4}$$

where $\mathbf{X}^+$ is the Moore-Penrose inverse of $\mathbf{X}$ and $\mathbf{X} \circ \mathbf{Y}$ is the Hadamard Product $(\mathbf{X} \circ \mathbf{Y})_{ij} = A_{ij}B_{ij}$ (an element-wise product). Next, we calculate the new concentration matrix as the projection of the data onto the basis set

$$\mathbf{C}' = \left(\mathbf{G}^+\mathbf{A}\right)^T . \tag{5}$$

RAW allows the users to constrain one or more components to have positive concentration. If that constraint is used, it is applied to $\mathbf{C}'$ at this stage, setting all negative values in each constrained component to zero,

$$C'_{jk} := \begin{cases} C'_{jk} & \textit{if } C'_{jk} > 0 \text{ or component k unconstrained} \\ 0 & \textit{if } C'_{jk} < 0 \text{ and component k constrained} \end{cases}, \tag{6}$$

where := is the assignment operator. The columns of $\mathbf{C}'$ are then normalized by their area

$$C'_{jk} := C'_{jk} / \left(\sum_h C'_{hk} M_{hk}\right). \tag{7}$$

The final step in the iteration is to test convergence. We calculate the absolute change in $\mathbf{C}$ as

$$\delta = \sum_{jk} \left| C_{jk} - C'_{jk} \right|, \tag{8}$$

and if $\delta < \delta_0$ the iterative algorithm has converged. If the algorithm has not converged, and the current iteration is less than the maximum number of iterations, we repeat the iterative step, first assigning $\mathbf{C} := \mathbf{C}'$, otherwise we exit the algorithm without a solution.

In the case where the algorithm has converged, we assign $\mathbf{C} := \mathbf{C}'$ then calculate the scattering profiles and errors as follows. Note that no matter which approach is used to find $\mathbf{C}$ we calculate the profiles and errors the same way. First, we calculate a set of coefficients $\mathbf{B}$,

$$\mathbf{B} = \left(\left(\mathbf{M} \circ \mathbf{C} \right)^T \right)^+, \tag{9}$$

from which the scattering profiles of the pure components can be computed as

$$J_k(q_i) = \left(\mathbf{DB} \right)_{ik}, \tag{10}$$

where $\mathbf{D}$ is the matrix of original intensities defined in S2.1.1. The uncertainties are then calculated as

$$\Delta J_k(q_i) = \sqrt{\left(\left(\mathbf{E} \circ \mathbf{E} \right) \left(\mathbf{B} \circ \mathbf{B} \right) \right)_{ik}}, \tag{11}$$

where $\mathbf{E}$ is the matrix of original uncertainties defined in S2.1.1.

S2.1.3. Explicit rotation method

An alternative approach is to explicitly compute the concentration matrix. We start by defining a reduced matrix of scattering profiles $\bar{\mathbf{A}}$ as

$$\bar{\mathbf{A}} = \bar{\mathbf{U}} \bar{\mathbf{S}} \bar{\mathbf{V}}^T, \tag{12}$$

which is the matrix constructed from the first n_s singular values and vectors. We then assume that our matrix $\bar{\mathbf{A}}$ can be represented as the product of a matrix with the pure component profiles, $\mathbf{I}$, times the concentration matrix,

$$\bar{\mathbf{A}} = \mathbf{IC}. \tag{13}$$

Equating these, and left multiplying by $(\bar{\mathbf{U}}^T\mathbf{I})^{-1}\bar{\mathbf{U}}^T$ gives

$$\mathbf{C} = (\bar{\mathbf{U}}^T\mathbf{I})^{-1}\bar{\mathbf{S}}\bar{\mathbf{V}}^T = \mathbf{T}\bar{\mathbf{V}}^T \ , \tag{14}$$

where we have defined the transformation matrix $\mathbf{T}$. This matrix is unknown, but we can solve for it using the information obtained from the evolving factor plots, $\mathbf{p}^{(f)}$ and $\mathbf{p}^{(b)}$.

Consider the i-th row of $\mathbf{C}$, $\mathbf{c}_{i,:}$,

$$\mathbf{c}_{i,:} = t_{i,:}\bar{\mathbf{V}}^T \ . \tag{15}$$

Recall that we have constrained $\mathbf{c}_{i,:}$ to be zero outside of the range defined by $\mathbf{p}_i^{(f)}$ and $\mathbf{p}_i^{(b)}$. Taking only the portion of the vectors and matrix outside of that range, we can write

$$\mathbf{0} = \mathbf{t}_{i,:}\bar{\mathbf{V}}_0^T \ , \tag{16}$$

where $\bar{\mathbf{V}}_0^T$ is the matrix made by removing the columns j of $\bar{\mathbf{V}}^T$where $\mathbf{p}_i^{(f)} \le j \le \mathbf{p}_i^{(b)}$. This equation has a trivial solution, $\mathbf{t}_{i,:} = \mathbf{0}$, but as $\bar{\mathbf{V}}_0^T$ is not full rank it also has a non-trivial solution. Because $\bar{\mathbf{V}}_0^T$ is not full rank, we are free to pick a component as an arbitrary value, in this case we make the first component of $\mathbf{t}_{i,:}$ equal 1. This gives

$$\mathbf{0} = 1 * \bar{\mathbf{V}}_{0\ 1,:}^T + \mathbf{t}_{i,2:n_s}\bar{\mathbf{V}}_{0\ 2:n_s,:}^T \ . \tag{17}$$

Note that the n_s limit of the slices is the full size of the matrix, but is included for clarity. This can be solved for $\mathbf{t}_{i,2:n_s}$,

$$\mathbf{t}_{i,2:n_s} = -\bar{\mathbf{V}}_{0\ 1,:}^T\left(\bar{\mathbf{V}}_{0\ 2:n_s,:}^T\right)^+ \ . \tag{18}$$

In combination with the picked first value of 1, this gives the full $\mathbf{t}_{i,:}$.

The complete transformation matrix $\mathbf{T}$ can be found row by row in this fashion, and then the concentration matrix $\mathbf{C}$ found via equation (14). Note that using this approach the concentration matrix may be negative, in which case multiply by -1. The concentration matrix here is actually the transpose of that defined in Section S2.1.2 Once the concentration matrix is found, the transpose is taken and then the scattering profiles are calculated as in the last part of Section S2.1.2 (equations (9) to (11)).

Note that in the trivial case of one component, this approach does not work. However, in that case no deconvolution is necessary.

The strength of this method is that it is fast, and it (almost) always returns a result. However, in our experience the results are not always as good as the iterative method, possibly because we cannot impose the extra constraint of positive concentration.

S2.1.4. Hybrid rotation method

The hybrid method combines the iterative and explicit methods. It works exactly the same as the iterative method described in S2.1.2, except for the generation of the initial concentration matrix $\mathbf{C}$. As with the iterative method, if a successful rotation has already been performed, it uses that as the initial matrix $\mathbf{C}$. When that is not the case, it uses the explicit method to generate the initial matrix $\mathbf{C}$. This usually gives significantly quicker convergence for the method, though the final results are the same. We generally recommend the use of the hybrid method.

S2.2. Validation of EFA implementation relative to the original

In order to ensure our implementation of the EFA method matched that described in (Meisburger et al., 2016), the original data and Matlab (The MathWorks) scripts used in that paper were obtained from Dr. Meisburger. Using the same buffer subtraction, total frame range, component limits, and convergence settings (not constraining C>0, 10000 iterations, iterative mode), we obtained essentially identical results, as shown in Figure S6. The scattering profiles overlap at all *q*, and the R_g values agree to two decimal places. We suspect that the minor differences (most visible in the high *q* region) are due to differences in underlying numerical methods implemented in numpy and Matlab.

S2.3. Our experiences on the practical limitations of EFA

The strength of EFA is that it is model independent, that is, there are no assumptions about the peak shape (such as the Gaussian and modified Gaussian shapes used in US-SOMO (Brookes et al., 2013, 2016)) or scattering profile (such as a linear Guinier region assumed in DELA (Malaby et al., 2015)). This makes it complimentary to the other methods, and able to provide independent validation of the deconvolved scattering profiles. However, it does have practical limitations (which may improve as the method becomes more refined). Based on our experience we have found:

1) EFA works best with flat baseline regions on both sides of the region to be deconvolved. If it is not clear that all singular values have returned to baseline, the deconvolution is more prone to failure, and can have very large chi-squared values at the edges.

2) EFA works best on subtracted profiles.

3) As a consequence of the previous limitation, EFA is not suitable for deconvolution of measurements with a changing baseline, such as may be cause by capillary fouling.

Based on limited analysis of sample data sets, we have found that EFA yields broadly similar results to US-SOMO when deconvolving overlapping peaks. A detailed comparison of these two methods is beyond the scope of this paper.

S3. Supporting References

All references included in the sup are in the references section in the main paper.

S4. Supporting Figures

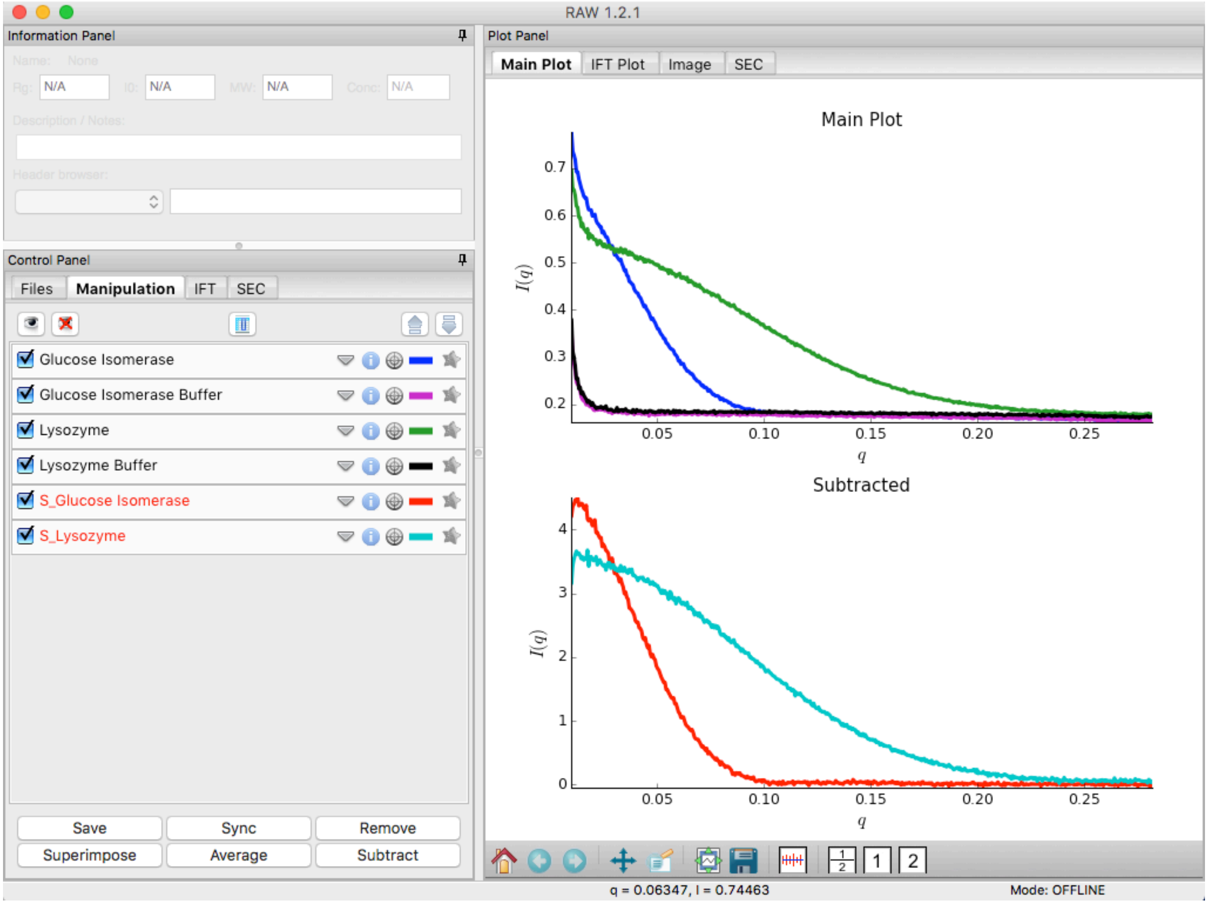

Figure S1 The manipulation panel and main plots in the RAW software with Lysozyme and Glucose Isomerase data loaded.

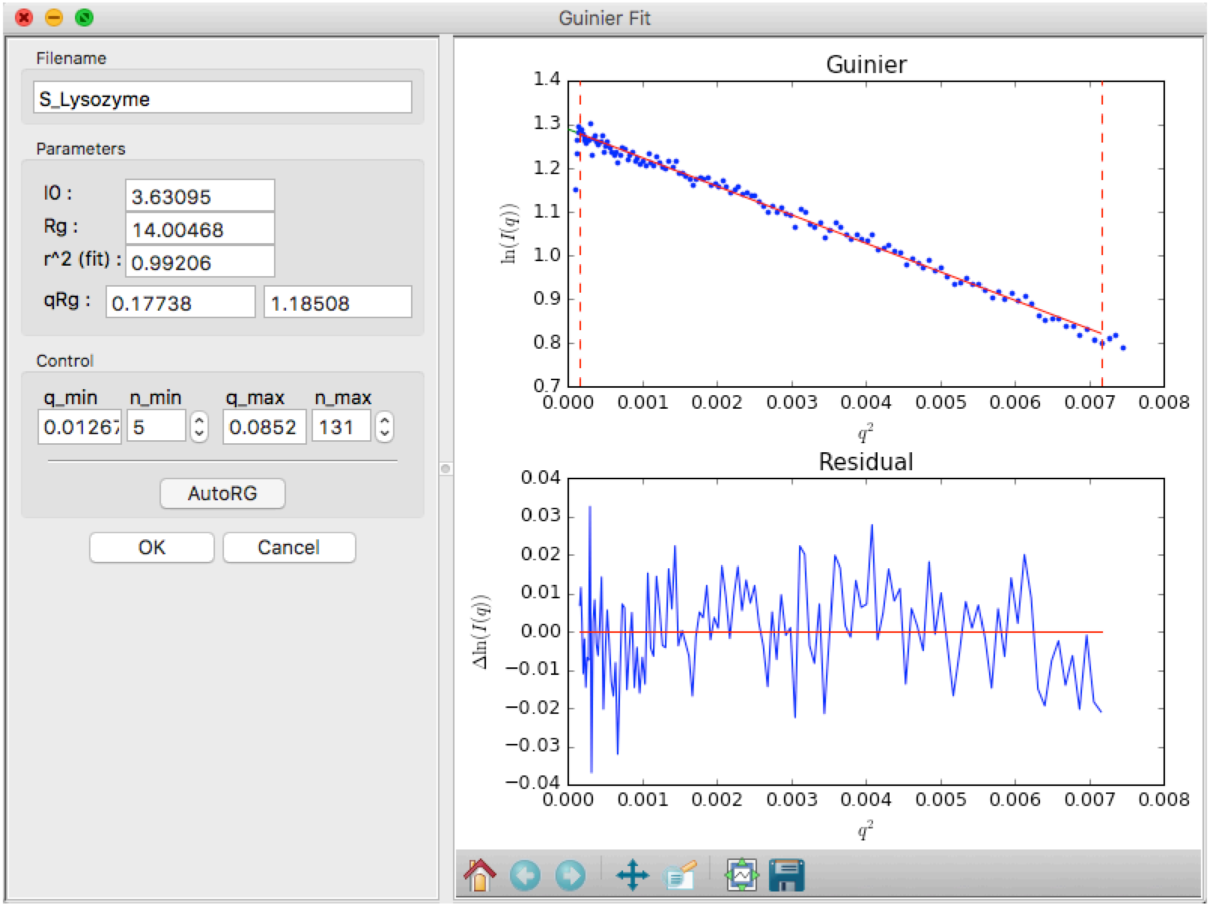

Figure S2 The Guinier fit window, which allows interactive and automated fitting.

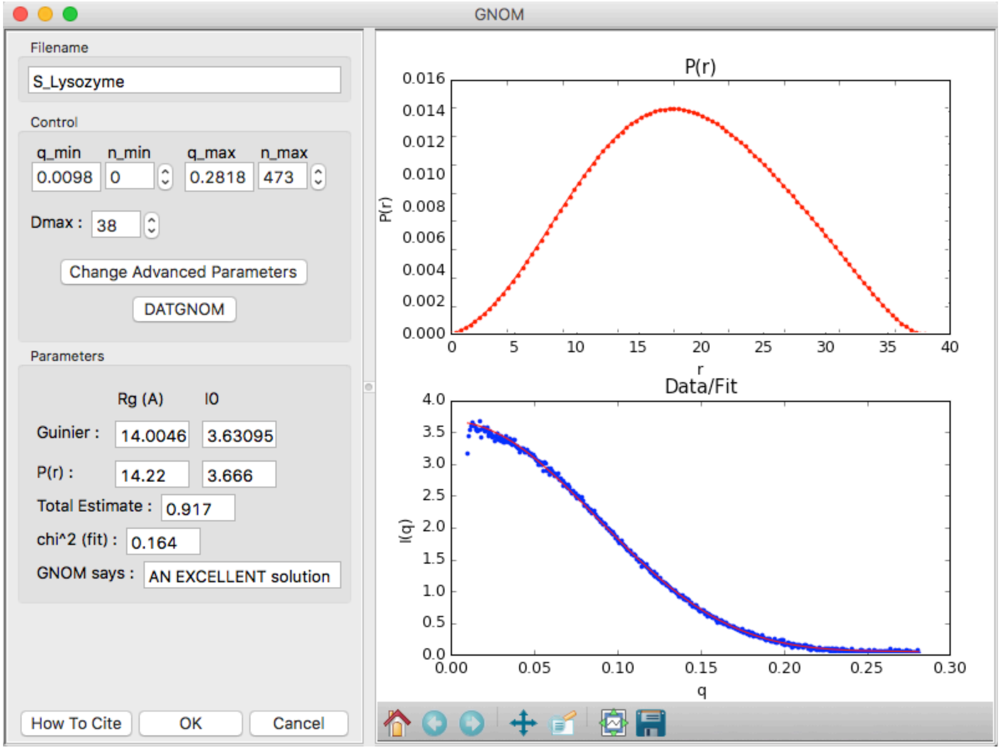

Figure S3 The GNOM window for determining IFTs by that method.

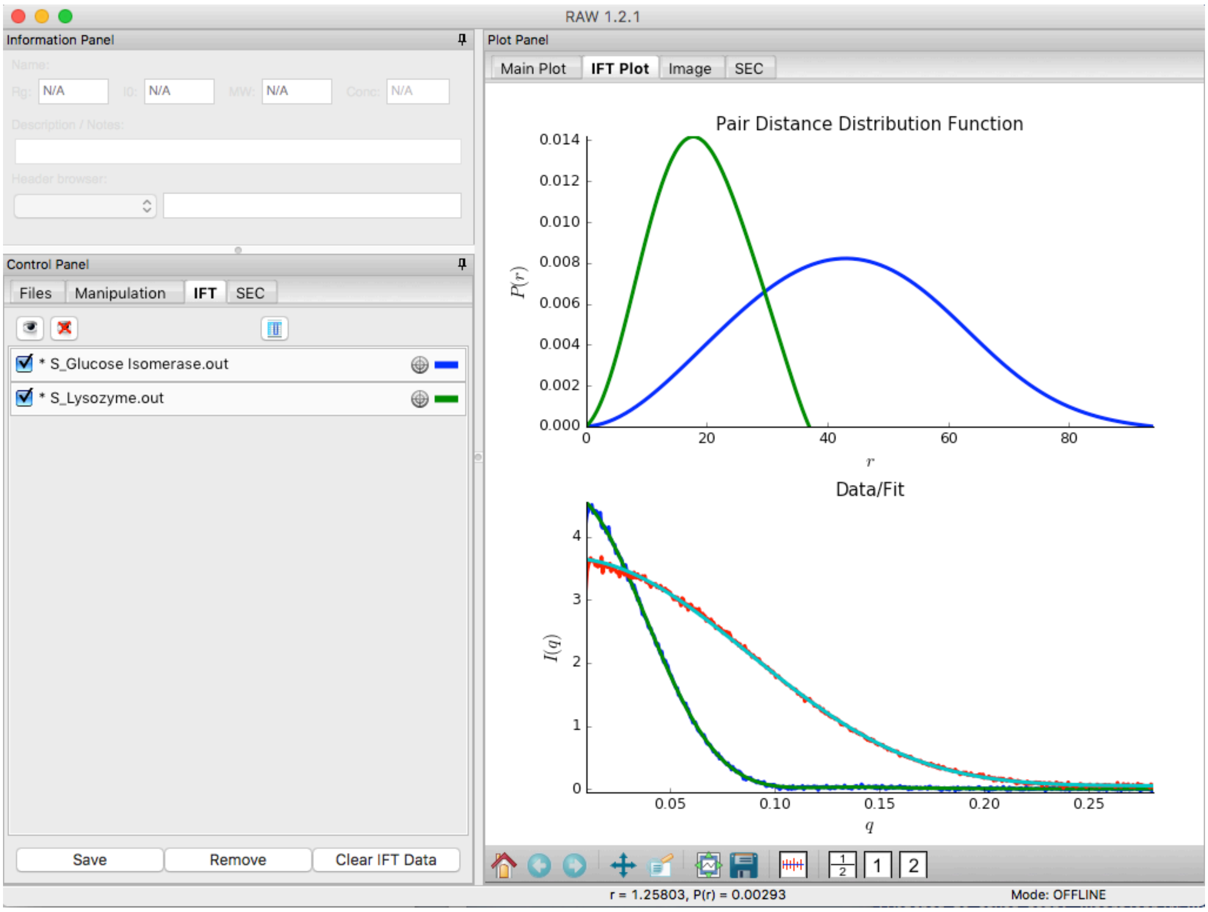

Figure S4 The IFT plot and control panel showing GNOM generated IFTs for Lysozyme and Glucose Isomerase.

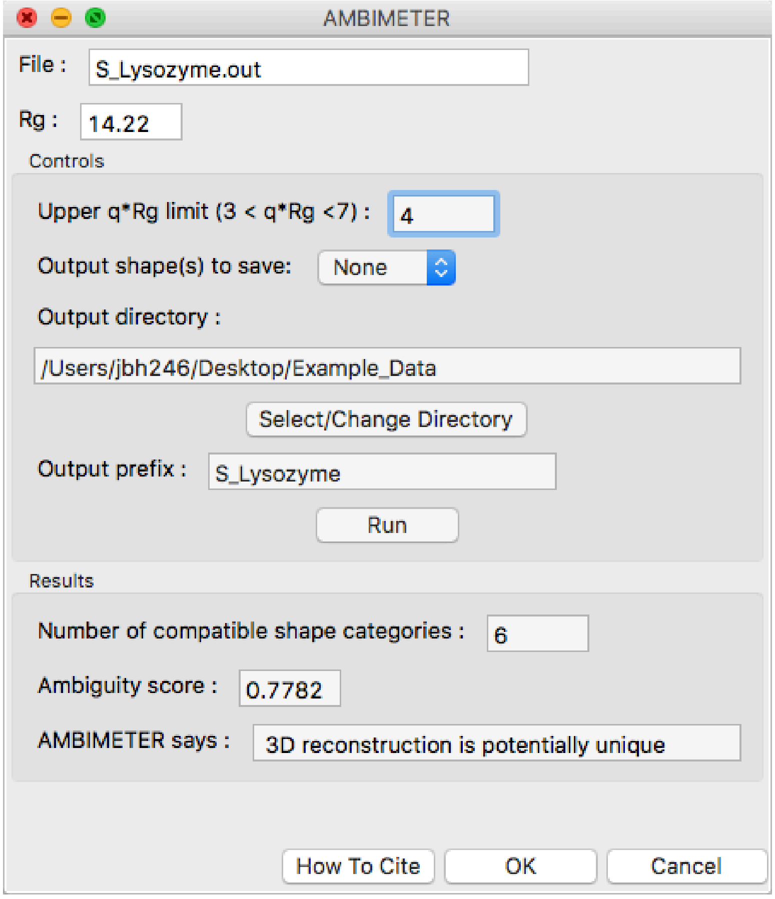

Figure S5 The AMBIMETER window, which shows the results of an ambiguity assessment of the scattering profile and allows the user to save the matching low resolution shapes from the assessment library.

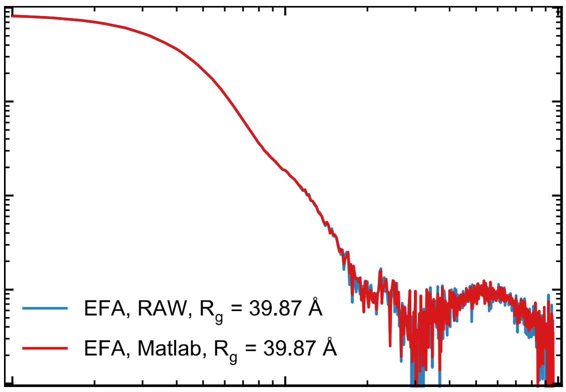

Figure S6 This figures shows scattering profiles extracted from overlapping SEC-SAXS data for phenylalanine hydroxylase. The blue curve (mostly hidden under the red curve) shows the EFA results obtained using the Matlab program described in (Meisburger et al., 2016), while the red curve shows the essentially identical results obtained in RAW. Additionally, the R_g for both curves agrees to two decimal places, further supporting that the results are functionally identical.

S5. Supporting Tables

Table S1 This table shows the molecular weight values calculated by the SAXS MoW2 online calculator and the adjusted Porod volume method of RAW. For protein database IDs that start with SAS, the scattering curves were taken from the SASBDB database (https://www.sasbdb.org/), the HS104P protein was taken from the BIOISIS database (http://www.bioisis.net/). For the HS104P protein the scattering profile labelled wtD1merged was used. All results given are for identical Guinier regions used in RAW and the SAXS MoW2 calculator (http://saxs.ifsc.usp.br/, accessed 27/03/2017). All results are for identical Guinier regions used in RAW and the MoW2 calculator.

Protein Database ID	Maximum q (Å^{-1})	MoW2 MW (kDa)	RAW MW (kDa)	Ratio RAW/MoW2
HS104P	0.20	866.4	860.3	0.9930
	0.30	861.8	855.4	0.9926
	0.40	841.7	837.4	0.9949
SASDAN7	0.20	491.6	491.5	0.9998
	0.30	463.4	464.0	1.0013
	0.40	430.4	430.8	1.0014
SASDA59	0.20	180.4	180.8	1.0022
	0.30	163.9	164.3	1.0024
	0.40	162.8	167.7	1.0301
SASDAK6	0.20	164.5	164.6	1.0006
	0.30	170.6	170.6	1.0000
	0.40	166.7	166.8	1.0006
SASDB26	0.20	90.5	90.5	1.0000
	0.30	77.6	77.7	1.0013
	0.40	69.9	70.1	1.0014
SASDBS6	0.20	61.6	61.7	1.0016
	0.30	53.3	53.3	1.0000
	0.40	49.7	49.7	1.0000
SASDBP4	0.20	28.9	28.6	0.9896
	0.30	25.6	25.3	0.9883
	0.40	23.6	23.4	0.9915

SASDAC2	0.20	8.6	8.6	1.0000
	0.30	8.8	8.8	1.0000
	0.40	8.5	8.6	1.0118

Table S2 This table shows the molecular weight values calculated by ScÅtter and RAW using the volume of correlation method. For protein database IDs that start with SAS, the scattering curves were taken from the SASBDB database (https://www.sasbdb.org/), the HS104P protein was taken from the BIOISIS database (http://www.bioisis.net/). For the HS104P protein the scattering profile labelled wtD1merged was used. All results given are for identical Guinier regions used in RAW and ScÅtter.

Protein Database ID	Maximum *q* (Å^{-1})	ScÅtter MW (kDa)	RAW MW (kDa)	Ratio RAW/ ScÅtter
HS104P	0.3	835.5	835.8	1.0004
	0.4	830.5	811.4	0.9770
SASDAN7	0.3	428.3	428.3	1.0000
	0.4	421.7	397.5	0.9426
SASDA59	0.3	160.5	160.4	0.9994
	0.4	158.7	153.0	0.9641
SASDAK6	0.3	143.6	143.6	1.0000
	0.4	142.6	137.5	0.9642
SASDB26	0.3	59.0	59.0	1.0000
	0.4	57.5	52.0	0.9043
SASDBS6	0.3	46.3	46.3	1.0000
	0.4	45.1	41.5	0.9202
SASDBP4	0.3	23.9	23.9	1.0000
	0.4	23.2	21.0	0.9052
SASDAC2	0.3	11.2	11.2	1.0000
	0.4	10.9	9.1	0.8349
